# Ladarixin, a dual CXCR1/2 inhibitor, attenuates experimental melanomas harboring different molecular defects by affecting malignant cells and tumor microenvironment

**DOI:** 10.18632/oncotarget.14803

**Published:** 2017-01-24

**Authors:** Daria Marley Kemp, Alyson Pidich, Mary Larijani, Rebecca Jonas, Elizabeth Lash, Takami Sato, Mizue Terai, Maria De Pizzol, Marcello Allegretti, Olga Igoucheva, Vitali Alexeev

**Affiliations:** ^1^ Department of Dermatology and Cutaneous Biology, Thomas Jefferson University, Philadelphia, PA, USA; ^2^ Department of Medical Oncology, Thomas Jefferson University, Philadelphia, PA, USA; ^3^ Dompé Farmaceutici SpA, Via Campo di Pile, LAquila, Italy

**Keywords:** CXCR1/2 inhibitor, melanoma, tumor growth inhibition, melanoma apoptosis, tumor microenvironment

## Abstract

CXCR1 and CXCR2 chemokine receptors and their ligands (CXCL1/2/3/7/8) play an important role in tumor progression. Tested to date CXCR1/2 antagonists and chemokine-targeted antibodies were reported to affect malignant cells *in vitro* and in animal models. Yet, redundancy of chemotactic signals and toxicity hinder further clinical development of these approaches. In this pre-clinical study we investigated the capacity of a novel small molecule dual CXCR1/2 inhibitor, Ladarixin (LDX), to attenuate progression of experimental human melanomas. Our data showed that LDX-mediated inhibition of CXCR1/2 abrogated motility and induced apoptosis in cultured cutaneous and uveal melanoma cells and xenografts independently of the molecular defects associated with the malignant phenotype. These effects were mediated by the inhibition of AKT and NF-kB signaling pathways. Moreover, systemic treatment of melanoma-bearing mice with LDX also polarized intratumoral macrophages to M1 phenotype, abrogated intratumoral *de novo* angiogenesis and inhibited melanoma self-renewal. Collectively, these studies outlined the pre-requisites of the successful CXCR1/2 inhibition on malignant cells and demonstrated multifactorial effects of Ladarixin on cutaneous and uveal melanomas, suggesting therapeutic utility of LDX in treatment of various melanoma types.

## INTRODUCTION

Malignant melanoma expresses and secretes various CXC chemokines, including CXCL1, CXCL2, CXCL3 (GRO family chemokines) and CXCL8 (IL-8). These molecules render the tumor microenvironment and facilitate progression and metastatic dissemination of melanomas *via* autocrine and paracrine activation of CXCR1 and CXCR2 chemokine receptors (reviewed in [1]). Studies on spontaneously immortal mouse melanocytes showed that stable expression of GRO family members enhances colony-forming abilities of the melanocytes, whereas antibody-mediated blocking of these chemokines inhibits experimental melanoma growth [2–4]. Elevated expression of CXCL8 (IL-8) was also associated with the intratumoral endothelial cell chemotaxis, neovascularization and angiogenesis *in vitro* and *in vivo* [5]. Up-regulated expression of IL-8 and CXCL1 were also associated with NF-kB transcription factor activity in cultured melanoma cells [6, 7]. All of these observations reinforce the concept that Gro-family chemokines and CXCL8 (IL-8) act as paracrine and autocrine mediators on melanoma growth and progression. The biological effects of these chemokines are mediated through two G protein-coupled receptors, CXCR1 and CXCR2 [8]. Engagement of these receptors induces intracellular signaling transmitted through heterotrimeric G proteins with Gαi being a predominant G protein coupled to this receptor family [9]. CXCR1 and CXCR2 receptors also exhibit a markedly distinct ligand binding pharmacology: CXCR1 is predominantly activated by CXCL8 and CXCL6, whereas CXCR2 could be activated by CXCL1-3 and 5-8 [10]. Expression of both receptors and multiple ligands by melanoma present certain challenges in designing therapeutic strategies to attenuate the effects of these chemokines [11].

To date, several strategies were employed to reduce/inhibit intracellular signaling mediated by CXCR1 and CXCR2 receptors. A number of ligand-blocking antibodies and small molecular weight antagonists of these chemokines, particularly CXCL8, were developed and tested [11]. Although blocking of individual chemokines provided certain benefits in treatment of acute and chronic inflammation [12], this approach may not provide desired outcome in treating neoplasms due to the redundancy of chemotactic signals.

Further search for effective competitive antagonists led to the identification of a number of compounds that can block CXCR1/2 receptor [11]. An advanced development program was originated by Dompé Farmaceutici with Reparixin, the first non-competitive allosteric CXCR1/2 inhibitor that is currently under active clinical investigation for the prevention of graft loss in pancreatic islet transplantation (Phase 3) and treatment of metastatic triple negative breast cancer (Phase 2). Ladarixin (LDX) is a second generation dual CXCR1/2 inhibitor due to its > 100 fold higher affinity for the CXCR2 receptor and improved pharmacokinetic properties that make it suitable for oral chronic administration. Ladarixin inhibits human polymorphonuclear leukocyte (PMN) migration to CXCL8 (IC50 at 0.7 nM) *in vitro*, and prevents PMN infiltration and tissue damage in several models of IR injury *in vivo* [13]. LDX is well-tolerated at all studied doses and showed excellent safety profile in human subjects in current clinical trials for the treatment of Type 1 diabetes (unpublished data). In this study, we demonstrated that LDX attenuates progression of different melanoma types *in vivo* *via* inhibition of cell cycle progression and motility, blocking of the pro-survival intracellular signals and induction of apoptosis, alteration of the intratumoral recruitment of the endothelial cells and *de novo* angiogenesis, and hindering of the melanoma self-renewal mechanisms.

## RESULTS

### Analysis of CXCL1/2/3/8 chemokines and CXCR1/2 receptors in primary melanoma cells

**Figure 1:**
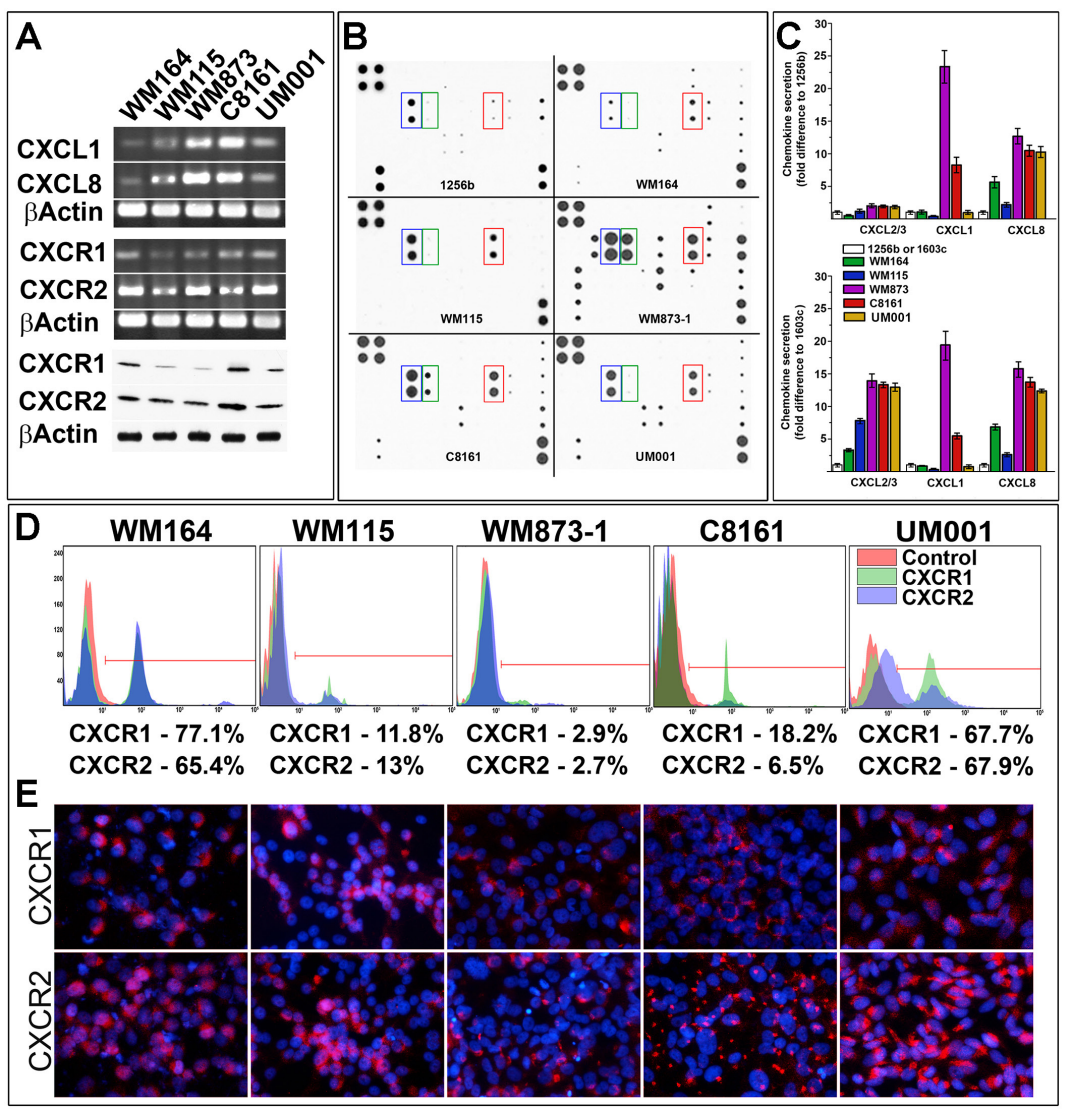
Analysis of chemokines and receptors in primary melanoma cells **A**. RT-PCR and Western Blot analyses of CXCL1/8 chemokines and CXCR1/2 receptors expression in different melanoma cell lines (as indicated on the panel). **B**. Representative images of the antibody array analysis used for the detection of the secreted chemokine. Blue frame - CXCL1/2/3 (Gro family) chemokines; Green frame - CXCL1; Red frame - CXCL8. **C**. Quantitation of chemokines secretion by melanoma cells relative to light-pigmented (1603c) and dark-pigmented (1256b) melanocytes. Data collected from 3 independent measurements and presented as a fold difference ± SD. Cell lines are indicated on the panel. **D**. Analysis of cell-surface CXCR1/2 in melanoma cells by FACS. Cell lines and receptors are indicated on the histograms. Calculated percentage of receptor-positive cells indicated below the histograms. **E**. Detection of CXCR1/2 receptors by fluorescently-labeled receptor-specific antibodies. Red - CXCR1/2 receptors, Blue - DAPI nuclear staining.

Considering heterogeneity of human melanomas, various molecular defects associated with discrete types of this neoplasm, and variable patterns of chemokine/receptor expression, we examined several primary human melanoma cell lines characterized by different molecular defects for the expression of CXCR1/2 and their ligands (CXCL1/8). Cutaneous melanoma cells expressing mutant BRAF^V600E^(WM164, WM115, WM873) [14, 15], cells with non-defined molecular defect expressing BRAF^G464E^ and KRAS^G12D^ (C8161) [16] and uveal melanoma cells harboring an activating mutation in GNAQ^Q209P^ (UM001) [17] were used for this assessment. RT-PCR analysis showed that CXCL1 and CXCL8 were differently expressed in the analyzed cells with the overall lowest expression in WM164 and WM115 and the highest in WM873-1and in C8161 melanoma cells (Figure 1A). Secretion of chemokines from these cells and from primary dark- and light-pigmented primary human melanocytes (1256b and 1603c, respectively) assessed by chemokine antibody array showed that levels of CXCR1/2 ligands were higher in all but one (WM164) melanoma lines as compared to melanocytes (Figure 1B, 1C). Secretion of CXCL8 was consistently elevated in all malignant cells (Figure 1B, 1C). Expression CXCR1/2 receptors was also examined. RT-PCR and Western Blot analyses confirmed expression of both receptors in malignant cells with on average 2 times higher expression of CXCR2 as compared to CXCR1 (Figure 1A). However, FACS analysis demonstrated that CXCR1 and CXCR2 were not uniformly present on the surface of the malignant cells. More than 50% of WM164 and UM001cells have these receptors consistently present on the cell surface, whereas only small populations of WM873, C8161and WM115 were recognized as receptor-positive (Figure 1D). FACS also revealed the existence of a discrete population of the CXCR1/2 cell surface-positive malignant cells (Figure 1D). These observations were confirmed by the direct (Figure 1E) and indirect (Supplementary Figure S1A) immunofluorescent detection of the receptors showing that WM115 and WM873 cells contained receptor-positive and negative populations, whereas a majority of the WM164 and UM001 were positive for both receptors on the cell surface (Figure 1E, Supplementary Figure S1A).

### Ladarixin-mediated inhibition of melanoma motility, adhesion and survival *in vitro*

**Figure 2:**
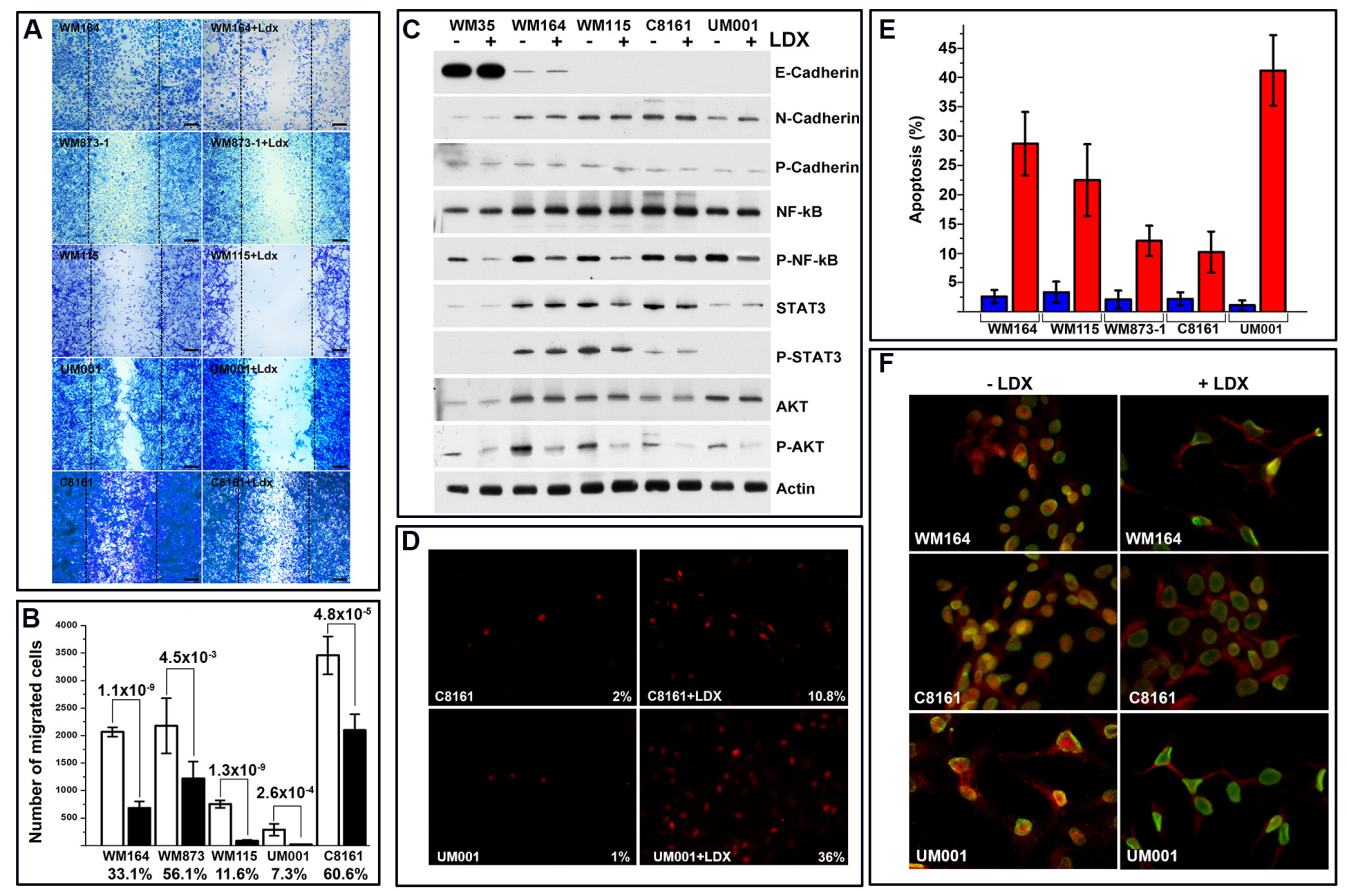
Analysis of melanoma cell motility and apoptosis **A**. Micrographs illustrating LDX-mediated inhibition of melanoma cell motility assessed by scratch assay. Cell lines and conditions indicated on the micrographs. Scale bar – 100 mm. Introduced scratches are outlined by puncture lines. **B**. Quantitation of LDX treated and control melanoma cell migration. Data is presented as an average number of cells migrated into the scratch (10 independent microscopic fields) ± SD. Statistical significance (p-value) was calculated using 2 tailed *t*-test is shown above the columns. Percentage of migrated LDX-treated cells relative to mock-treated control is shown below the columns. **C**. Western blot analysis of adhesion and signaling molecules in representative LDX-treated and control melanoma cells (indicated above the panels). Analyzed molecules are shown to the right of the panels. Phosphorylated NF-kB, STAT3 and AKT are marked as P-proteins. Beta actin was used as loading control. **D**. Microscopic analysis of apoptosis in control and LDX-treated melanoma cells by TUNEL assay. Cell lines, treatment and calculated percentage of apoptotic cells shown on representative images. **E**. Quantitation of apoptosis as determined by the TUNEL assay in all investigated melanoma cell lines (indicated below the columns). Data is presented as average of 3 independent experiments ± SD. Five random microscopic fields were used for each treatment condition/cell line to calculate percentage. **F**. Indirect immunofluorescent analyses of NF-kB nuclear translocation/activation in control and LDX-treated representative melanoma cells. Treatment and cell lines are indicated on the panels. Red – Nf-kB, Green – LaminA/C (outlines nuclear membrane).

Considering involvement of the CXCR1/2 signaling in migration, the effect of the LDX on motility of melanoma cells was examined using *in vitro* migration/scratch assay. The presence of LDX in normal culture media for 24 h led to an inhibition of melanoma cell motility (Figure 2A, 2B). A more profound inhibition was observed on cells with higher cell surface CXCR1/2 and lower expression of their ligands (WM164, WM115, and UM001 lines). Treatment of these cells with LDX led to 10-fold inhibition of cell migration. Exposure of WM873 and C8161 cells with lower cell surface receptor expression to LDX led to a 2-fold decrease of motility (Figure 2B).

In these experiments, we observed an increased number of dislodged cells in the presence of LDX. This sparked further assessment of cellular attachment and adhesion by the modified McClay substrata binding assay as described in our prior studies [18]. The presence of the LDX during plating and for additional 4 h did not alter the adhesion of the LDX-treated and control cells, although a slight reduction in a number of adherent UM001 cells was observed (Table 1). Exposure of melanoma cells to LDX for 3 days affected mostly WM164 cells with 3 times more cells remaining on plastic substrata when cultured in the absence of the LDX. Exposure of cells to LDX for an additional 2 days had a more detrimental effect on all cell lines. On average, 3 times more cells remained attached to plastic substrata when cultured in the absence of LDX (Table 2). These findings suggest that LDX-mediated blocking of the CXCR1/2 signaling affects expression of the adhesion molecules or leads to the cell death and detachment. Therefore, expression of cadherins was analyzed by Western blot under referenced above conditions. High expression of epithelial cadherin (E-cadherin) was detected only in WM35 melanoma cells isolated from primary (radial growth phase) lesion. In cells from more advanced lesions (vertical growth phase or metastatic), a substantial reduction or absence of this cadherin was observed (Figure 2C). N-Cadherin, which is commonly associated with invasion and metastasis of neoplasms, was detected in all cell lines isolated from local and distal metastatic lesions with the lowest expression in the WM35 cell line. P-cadherin, whose expression is associated with poorly differentiated carcinomas [19, 20], was barely detectable in all examined cells. Treatment of cells with LDX did not alter expression of these cadherins (Figure 2C), suggesting that progressive loss of attached LDX-treated cells could be attributed to apoptosis. Based on the *in situ* cell death detection (TUNEL) assay, on average, about 3% of all control cells were identified as apoptotic whereas LDX treatment increased apoptosis up to 10% for C8161 and WM873 cells, 25 % for WM 164 and WM115 cells, and up to 40% for UM001 (Figure 2D, 2E).

**Table 1:** Melanoma cell adhesion at plating

Ladarixin	WM164	C8161	TSUM1	Time
Ldx-	0.366±0.085	0.641±0.073	0.126±0.034	4hr
Ldx+	0.288±0.058	0.518±0.063	0.144±0.038	4hr
Ldx-	0.349±0.064	0.521±0.0532	0.394±0.048	3hr
Ldx+	0.221±0.074	0.435±0.034	0.313±0.058	3hr
Ldx-	0.310±0.053	0.524±0.041	0.338±0.069	2hr
Ldx+	0.221±0.051	0.483±0.049	0.322±0.055	2hr
Ldx-	0.333±0.069	0.519±0.061	0.346±0.066	1hr
Ldx+	0.219±0.054	0.418±0.044	0.301±0.034	1hr

Data is presented as optical density measured at 570 nm (OD A_570_) reflecting attached cells.

**Table 2:** Assessment of cellular attachment to the plastic substrata (ODA570)

Time	Ladarixin	WM164	C8161	TSUM1
5 Days	LDX -	0.178±0.02	0.175±0.012	0.156±0.012
5 Days	LDX +	0.048±0.017	0.049±0.011	0.066±0.013
4 Days	LDX -	0.192±0.014	0.17±0.014	0.155±0.012
4 Days	LDX+	0.05±0.013	0.074±0.017	0.081±0.017
3 Days	LDX -	0.199±0.018	0.17±0.012	0.182±0.013
3 Days	LDX +	0.059±0.012	0.185±0.012	0.157±0.015

Data is presented as optical density measured at 570 nm (OD A_570_) reflecting density of the attached cells.

Given that NF-kB supports survival of the malignant cells *via* Bcl-2/Bcl-XL-dependent pathway and augments transcription of CXCR1/2 ligands [1], we investigated whether LDX treatment abrogates NF-kB expression and/or activation. To assess whether LDX-mediated CXCR1/2 blocking affects NF-kB activity, we generated several melanoma cell lines stably expressing NanoLuc^®^ luciferase under the control of the NF-κB response elements. Exposure of these cells to LDX (1 ηM, 100 ηM, 1µM) for 24 h *in vitro* led to reduction of NanoLuc in all examined cells confirming LDX-mediated down-modulation of the NF-kB activity (Supplementary Figure S1B). Exposure of all malignant cells to LDX for 72 h led to an intracellular re-distribution of NF-kB and its accumulation in the cytoplasm. It was particularly evident in WM164 and UM001 cells (Figure 2F). Western Blot analysis with serine^536^-phosphorylated NF-kB-specific antibodies confirmed the reduction of the NF-kB activation and nuclear translocation in LDX-treated cells. It was most prominent in WM35, WM164, WM115, and UM001, and less pronounced in C8161 cells (Figure 2C). As Akt plays an important role in controlling the balance between survival, apoptosis and NF-kB activation [21], we also assessed the status of this serine-threonine kinase. Based on the Western Blotting, treatment of the cells with LDX did not alter the expression of the AKT but markedly reduced its phosphorylation on Thr308 in all examined cell lines (Figure 2C). Persistent activation of STAT3 has been shown to mediate several oncogenic features in many types of cancers, including melanoma [22]. CXCL8 (IL-8) signaling has been implicated in STAT3 activation and nuclear translocation [23], suggesting that inhibition of CXCR1/2 may also lead to the inhibition of STAT3 in melanoma cells. However, Western Blot analysis showed a relatively low basal level of STAT3 expression in all malignant cells and insignificant changes in STAT3 phosphorylation after LDX treatment (Figure 2C).

Collectively, these *in vitro* studies demonstrated that exposure of malignant cells to LDX resulted in down- modulation of cell cycle progression, motility, Akt and NF-kB phosphorylation-dependent activation, and induction of apoptosis independently of molecular defects underlying tumorigenic phenotype. These studies also demonstrated that LDX-mediated inhibition of CXCR1/2 has the most profound effect on melanoma cells with higher level of cell-surface receptors and lower secretion of ligands (eg. WM164), and only partial effect on cells with lower number of cell surface receptors and higher secretion of ligands (eg. C8161).

### Ladarixin-mediated inhibition of melanoma xenografts *in vivo*

**Figure 3:**
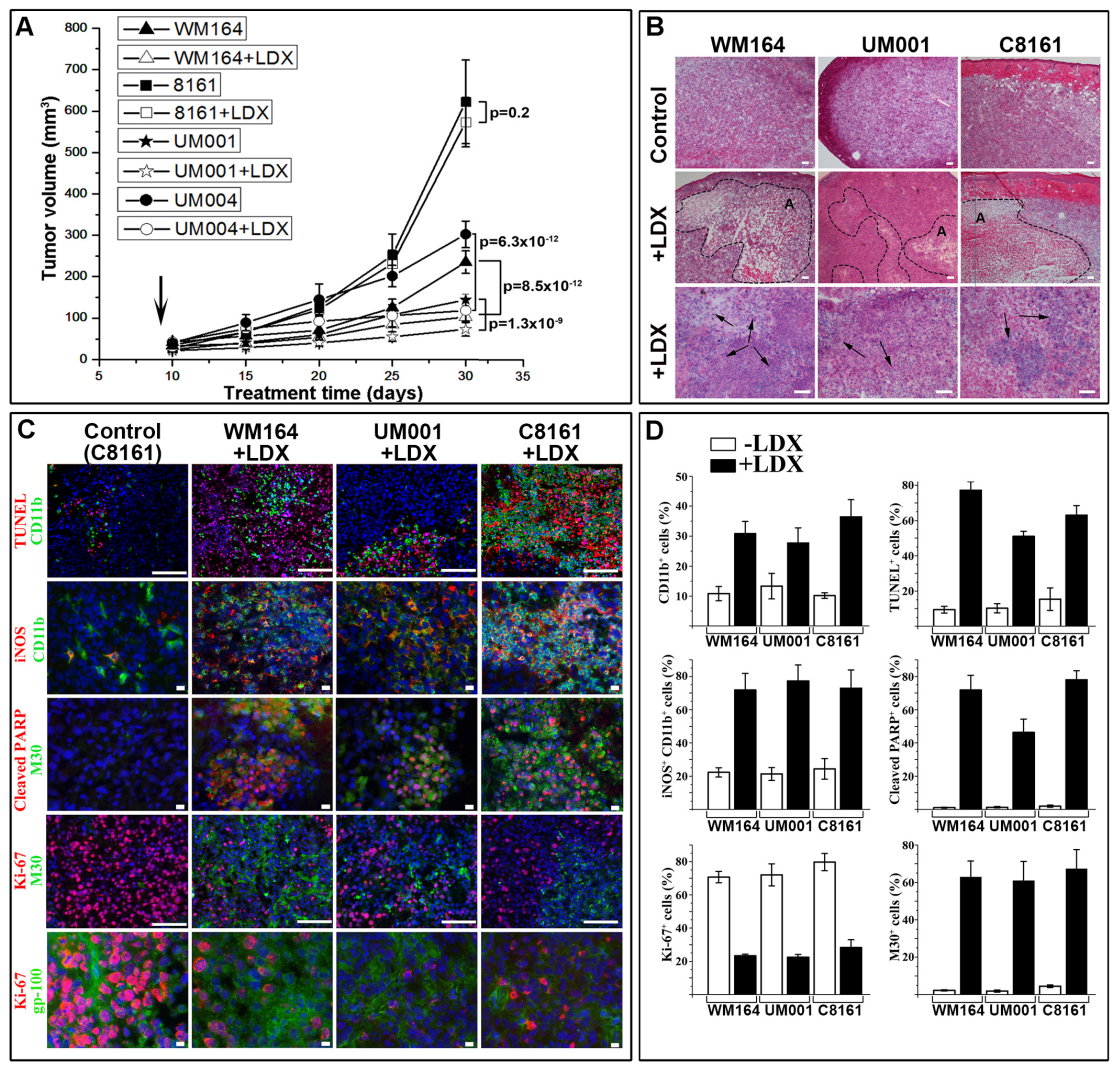
LDX-mediated inhibition of melanoma xenotransplants ***in vivo***. **A**. Graph of the lesions at 30 days showing tumor development in LDX-treated and control mice. Data is presented as a mean tumor volume (mm^3^) ±SD. Statistical significance is presented as p-value (shown of the graph). **B**. Representative micrographs of the H&E stained sections of the control and LDX treated lesions showing the presence of the apoptotic regions (dotted line) and leukocytic infiltrates (black arrows). **C**. Indirect immunofluorescence-based characterization of LDX-treated and control tumors. As all mock-treated lesions were similar, micrographs of the control C8161 lesions are shown as representative controls. Detected antigens are shown to the left of the panels in corresponding colors. Blue - DAPI nuclear staining. Scale bars: 10 µm (2, 3, and 5^th^ rows from the top) and 100 µm (1, 4^th^ rows from the top). **D**. Quantitation of antigen-positive cells shown on panel C. Data is presented as an average percentage of positive cells per microscopic field ± SD.

To evaluate the extent of LDX-mediated melanoma inhibition *in vivo* and better define the underlying molecular mechanisms, 4 different human melanoma cell lines characterized by various levels of cell surface CXCR1/2, ligands secretion and distinct molecular defects (WM164^V600E^,C8161, UM001^Q209P^ and UM004^Q209L^) were inoculated into nude athymic mice. Starting from day 10 after inoculation, when lesions reached about 50 mm^3^ (~4×4×3 mm), experimental groups (*n* = 10 per group) were receiving LDX *via* intraperitoneal (IP) injection once a day at 15 mg/kg. Control animals were receiving saline. Consistent with our *in vitro* studies, systemic administration of LDX had limited effect on growth of C8161 melanoma lesions but significantly inhibited growth of WM164, UM001 and UM004 melanomas (Figure 3A). All LDX-treated lesions were visually less vascularized (particularly UM001) compared to untreated controls (Figure 3A).

Histological examination showed that all lesions from LDX-treated animals contained apoptotic regions with elevated leukocytic infiltrate (Figure 3B). The largest apoptotic regions occupying on average 2/3 of the entire tumor volume were detected in LDX-treated C8161 lesions. These regions were characterized by the presence of TUNEL-positive melanoma cells and a large number of CD11b^+^ leukocytes (Figure 3C, 3D). Considering a dual role of the tumor-infiltrating CD11b^+^ macrophages that may prevent (M1 macrophages) or promote (M2 macrophages) tumorigenesis [24], expression of nitric oxide synthase 2 (iNOS) as a marker for the M1 macrophages was analyzed. Majority of the intratumoral CD11b^+^ cells in control lesions did not express iNOS (Figure 3C, 3D). On the contrary, more than 60% of tumor-infiltrating CD11b^+^ cells in LDX-treated lesions were iNOS-positive (Figure 3C, 3D).

Considering apoptosis as a primary mechanism involved in LDX-mediated melanoma cell death, we examined the status of the key apoptotic markers in LDX-treated and control lesions. Concurrent detection of caspase-cleaved PARP and cytokeratin 18 confirmed apoptotic mechanism of malignant cell death in all LDX-treated animals (Figure 3C, 3D). Quantitation of apoptotic cells showed that more than 60% of cells in the affected regions were positive for both apoptotic markers. A substantial reduction of the proliferating Ki-67^+^ cells and increased number of the cleaved cytokeratin 18 (M30+) cells were also observed in non-apoptotic regions (Figure 3C). Inverse correlation between Ki-67^+^ proliferating and M30^+^ apoptotic cell was most distinct in WM164 treated melanomas (Figure 3C, 3D). Immunofluorescent detection of Ki-67 and gp-100 melanoma differentiation antigen confirmed LDX-mediated inhibition of proliferation in malignant cell in non-apoptotic tumor regions (Figure 3C).

**Figure 4:**
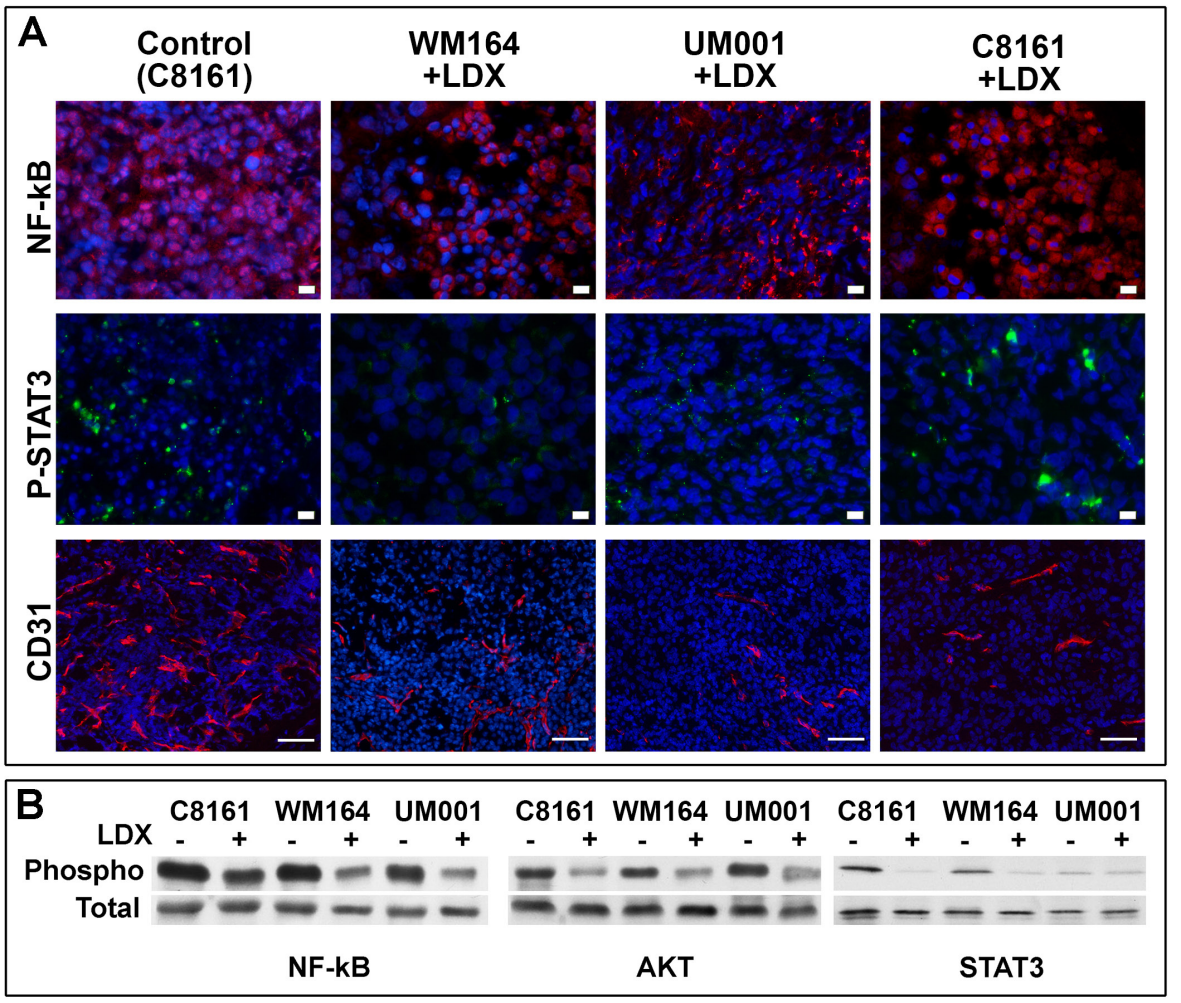
Analysis of NF-kB, STAT3, AKT and angiogenesis in control and LDX-treated lesions **A**. Indirect immunofluorescent analysis of NF-kB, STAT3 and CD31 (as a marker of angiogenesis *de novo*) in control and LDX-treated lesions. As all mock-treated lesions were similar, micrographs of the control C8161 lesions are shown as representative controls. Detected antigens (in corresponding colors) are show to the left of the panels. Blue - DAPI nuclear staining. Cell lines and treatments are shown above the panels. Scale bar - 10 µm. **B**. Western blot analysis of NF-kB, STAT3, AKT phosphorylation in control and LDX-treated lesions. Cell lines and treatments are shown above the panels.

To better define molecular mechanisms involved in LDX-mediated inhibition of melanoma *in vivo*, NF-kB and STAT3 in control and LDX-treated lesions were examined by the indirect immunofluorescence. This analysis demonstrated preferential nuclear localization of NF-kB in the control and cytoplasmic localization in treated lesions (Figure 4A). Detection of STAT3 in control and LDX-treated lesions did not reveal any significant differences, although a more pronounced nuclear staining of the STAT3 was detected in controls. These observations were further confirmed by Western blot analysis. A reduction of the Ser^536^-phosphorylated NF-kB and Thr^308^-phosphorylated AKT and Tyr^705^-phosphorylated STAT3 was observed in all LDX-treated lesions (Figure 4B), suggesting that LDX-mediated CXCR1/2 inhibition attenuates NF-kB/AKT activation making malignant cells susceptible to apoptosis and reducing their proliferative capacities.

Previous studies demonstrated that CXCR1/2 receptors are expressed by microvascular endothelial cells and that secretion of CXCR1/2 ligands by melanoma enhances *de novo* angiogenesis [25]. Indirect immunofluorescent detection of the CD31^+^ intratumoral blood vasculature demonstrated a drastic reduction of endothelial cell recruitment and formation of the intratumoral blood vessels in all examined LDX-treated lesions. Lack of the intratumoral angiogenesis was particularly evident in peri-apoptotic regions of the LDX-treated tumors, where blood vessels were not detected at all (not shown). A reduction CD31^+^ blood vessels in non-apoptotic regions of all LDX-treated lesions was apparent (Figure 4A) and confirmed by the assessment of microvessel density (Table 3).

**Table 3:** LDX-mediated inhibition intratumoral microvessel density (MVD)

Cells	MVD (± SD) - // + LDX	% inhibition	*t*-test
C8161	22±4 // 8±3	62	<0.05
WM164	18±4 // 4±2	78	<0.05
UM001	15±2 // 4±2	73	<0.05
UM004	14±2 // 5±2	65	<0.05

Immunofluorescent staining for microvessel using anti-CD31 was analyzed as described in Materials and Methods. MVD was quantitated on 10 independent microscopic fields per sample.

**Figure 5:**
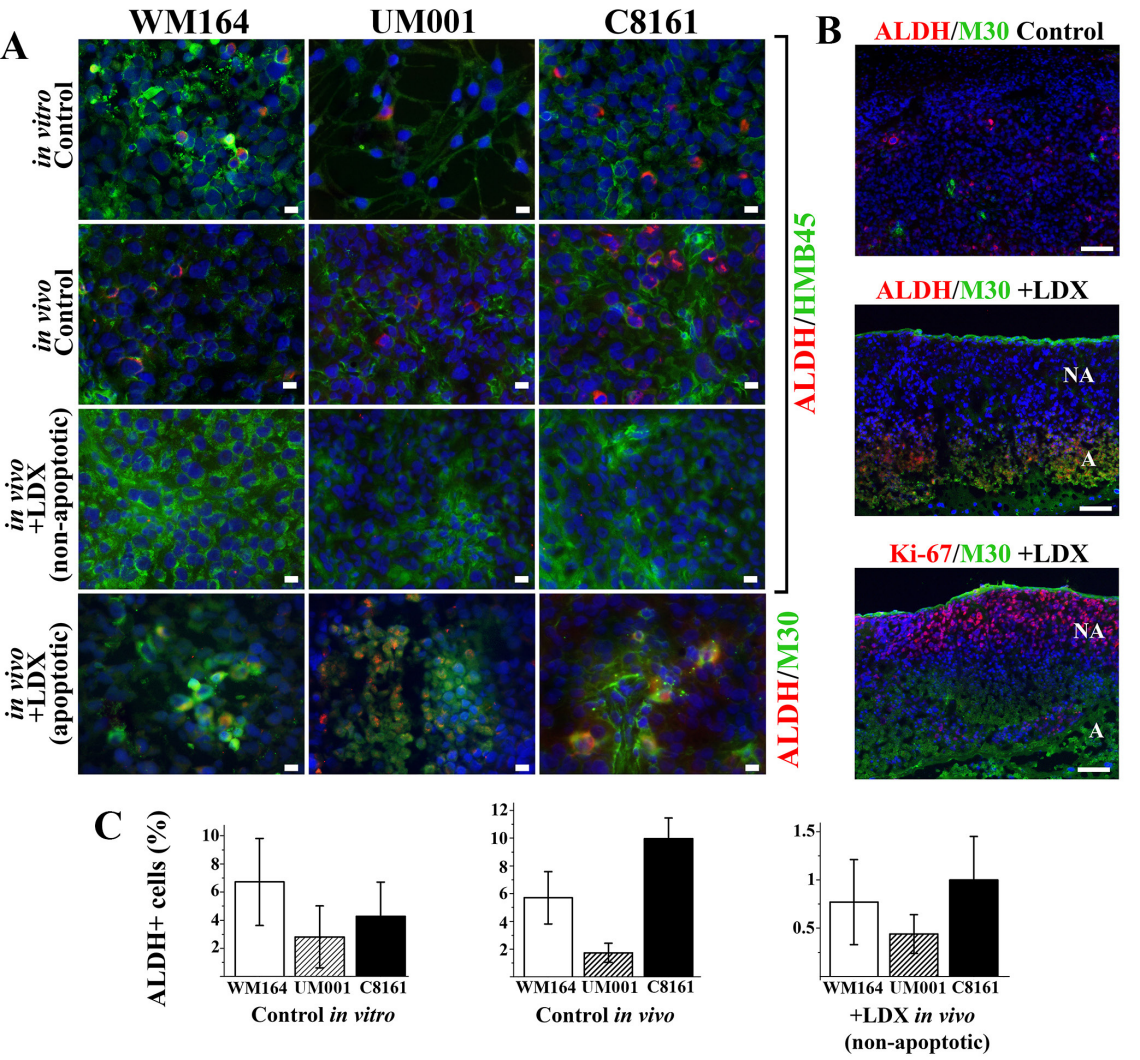
Assessment of tumor-initiating ALDH + melanoma cells ***in vitro*** and ***in vivo***. **A**. Indirect immunofluorescent detection of ALDH+ melanoma cells *in vitro* and *in vivo*. Detected antigens (in corresponding colors) are shown to the right of the panels. Scale bar - 10 µm. **B**. Indirect immunofluorescent detection of the ALDH^+^ cells in non-proliferating (Ki-67-negative), apoptotic (M30-positive) cells in LDX-treated lesions. Scale bar - 100 µm. **C**. Quantitation of ALDH^+^ cells in control cultured cells and non-apoptotic regions of the LDX-treated lesions (Panel A). Data is presented as average percentage of ALDH^+^ cells per microscopic field ± SD.

Several recent studies suggested that so called tumor-initiating cells responsible for melanoma self-renewal display high aldehyde dehydrogenase (ALDH) activity and enhanced tumorigenicity over ALDH-negative cells [26, 27] . Considering a potential correlation between CXCR1/2 expression and ALDH activity reported in breast carcinoma [28, 29], we tested how LDX treatment affects ALDH^+^ melanoma cells. In all cultured cells, independently of the tumorigenic mutation, about 4% of cells were ALDH-positive whereas in xenotransplants it was increased up to 10% in the lesions (Figure 5A, 5B). Treatment of tumor-bearing mice with LDX led to an inhibition of ALDH expression in non-apoptotic regions of the lesions where ALDH^+^, HMB45^+^ (gp100^+^) malignant cells were rarely detected (Figure 5A, 5B, 5C). Percentage of these cells decreased on average 4 times for UM001, 8 times for WM164, and 10 times for C8161 (Figure 5C). Concurrently, a higher percentage of the ALDH^+^ cells was detected in non-proliferating, apoptotic, M30^+^ regions of LDX treated lesions (Figure 5B, 5C). In fact, all ALDH^+^ cells were also positive for caspase-cleaved cytokeratin 18 (Figure 5B). Collectively, these data demonstrated that treatment of melanoma-bearing mice with LDX leads to the inhibition of ALDH in non-apoptotic malignant cells and its induction in cells undergoing apoptosis.

## DISCUSSION

Previous studies demonstrated that different CXCR1 and CXCR2 antagonists can block these receptors inhibiting inflammation and tumor growth in various animal models. For example, G31P, an IL-8 analog, was shown to block neutrophil infiltration, pyrexia, and pulmonary vascular pathology in endotoxemic animals [30, 31]. SCH-527123 and SCH-479833, dual CXCR1/2 and CXCR2 antagonists, were shown to inhibit migration and proliferation of A375SM melanoma cells [32]. SCH-527123 was described as a potent inhibitor of CXCR1- (IC50 = 41 nM) and CXCR2 (IC50 = 3 nM) mediated chemotaxis with high affinity [33]. It was shown to inhibit neutrophil recruitment, mucus production and goblet cell hyperplasia in animal models of pulmonary inflammation [34]. The potential therapeutic effect of SCH-527123 in the treatment of chronic pulmonary diseases has been widely investigated in several clinical studies, but the lack of evident long-term clinical benefit and associated neutropenia led to discontinuation of its clinical development [35–37]. In this study, we assessed therapeutic aspects of a novel, dual small molecule CXCR1/2 inhibitor, Ladarixin, to attenuate development and progression of human melanomas characterized by different molecular defects. Ladarixin is orally bioavailable, potent and selective non-competitive allosteric CXCR1/2 inhibitor that binds an allosteric pocket of the trans-membrane region of both receptors. The allosteric action results in a dramatic reduction of CXCR1 (IC50 = 0.9 nM) and CXCR2 (IC50 = 0.8 nM) mediated chemotaxis in the absence of any significant influence on ligand/receptor binding [38]. Ladarixin was also effective in decreasing CXCL8-induced polymorphonuclear leukocyte infiltration in several animal models without a significant dose-related reduction in systemic neutrophil counts. Confirmation of this last observation from Phase 1 clinical studies suggests that allosteric modulation may represent a promising approach to the design of safe and well-tolerated drugs acting at chemokine receptors.

Limited number of reports on targeted inhibition of Gro family chemokines, CXCL8 (IL-8) and their receptors (CXCR1 and CXCR2) in various tumor models including melanomas, suggested a potential applicability of these approaches for tumor growth inhibition (11). However, redundancy of chemotactic signals presents a significant challenge in designing therapeutic strategies to attenuate the effects of CXCR1/2 signaling on melanoma cells and tumor microenvironments, and the pre-requisites for the successful treatment remain undefined. Presented here analysis demonstrated that melanoma cells secrete different levels CXCL1 and CXCL8 and rather high levels of CXCL2 and CXCL3 chemokines (Figure 1). Concurrently, tumor cells express both CXCR1/2 receptors. FACS analysis revealed the existence of distinct populations of receptor-positive and receptor-negative cells in otherwise homogeneous malignant cell cultures (Figure 1D). This analysis also demonstrated that despite a rather high level of CXCR1/2 expression in some melanoma cells (eg. WM873 and C8161), only a small percentage of cells had receptors on the cell surface, whereas other cells (eg. WM164 and UM001) had a consistently high number of receptor-positive cells. These observations suggest that in malignant cells with high secretion of the autocrine chemokines and high level of CXCR1/2 expression (as in WM873 and C8161) a rapid binding and internalization of the ligand/receptor complexes may lead to the “removal” of the receptors from the cell surface, as previously observed for CXCR4-CXCL12 [39]. These findings also suggest that CXCR1/2 blocking may have limited direct effect on melanoma cells with the lower number of cell surface receptors and high levels of autocrine chemokines.

Consistent with the prior data obtained on A375SM cells using SCH-527123 inhibitor (29), LDX treatment inhibited melanoma cells motility. However, our analysis clearly demonstrated that LDX-mediated inhibition of motility, proliferation, AKT and NF-kB activation and induction of apoptosis directly correlated with the percentage of CXCR1/2-positive cells. Thus, WM164, WM115 and UM001 cells were more susceptible to the LDX treatment than WM873 or C6181 cell. Importantly, treatment of different melanoma cells *in vitro* with 1µM LDX was more effective than treatment of A37SM with 250 µM SCH-527123 (29).

Consistent with the *in vitro* data, treatment of the experimental melanoma-bearing mice with LDX (15 mg/kg) led to the inhibition of tumor growth independently of the molecular defects underlying tumorigenic phenotype. Thus, statistically significant inhibition of BRAF^V600E^ WM164, GNA11^Q209L^ UM001 and GNAQ^Q209P^ UM004 melanomas was observed (Figure 3). These observations support the notion that malignant cells with higher cell-surface CXCR1/2 expression and lower expression the ligands (e.g. WM164) are more susceptible to LDX than cells with lower sell surface receptors (e.g. C8161). Although, treatment of C8161 melanoma-bearing mice with LDX did not alter outgrowth of the intradermal tumors, normalized rate of apoptosis and inhibition of proliferation were similar in all LDX-treated lesions (Figure 3D) and C8161 lesions contained large apoptotic regions approaching 2/3 of the entire tumor volume. Such discrepancy could be explained by faster proliferation of the C8161 cells, higher expression of the CXCR1/2 ligands that cause rapid internalization of the receptors and higher microvascular density, particularly, in the periphery of the established C8161 tumors. Together with the inhibition of AKT and NF-KB activation/phosphorylation *in vitro* (Figure 2) and *in vivo* (Figure 4A), our data indicate that LDX-mediated induction of apoptosis in malignant cells is associated with down-modulation of the AKT/NF-kB-mediated pro-survival signals. Comparison of our *in vivo* data with previous findings obtained using SCH-527123 at 100 mg/kg dose [32] showed that systemic treatment of tumor-bearing mice with LDX is more effective, as reflected by the inhibition of melanoma cell proliferation, angiogenesis and induction of apoptosis (Figure 3D, Figure 4).

Moreover, we observed that apoptotic regions in LDX-treated lesions were heavily infiltrated with CD11b^+^ TAMs characterized by the uniform expression of the iNOS, a well-known maker of the M1 macrophages with tumoricidal activity. These findings suggest that LDX treatment may directly or indirectly affect polarization of the TAMs contributing further to the inhibition of the neoplasms.

Previous studies demonstrated that CXCR1/2 expressed on the surface of the blood endothelial cells play an important role in the recruitment and the intratumoral *de novo* angiogenesis [5, 25]. Our analysis demonstrated a significant reduction of the intratumoral CD31^+^ endothelial cells and blood vessels in LDX-treated tumor-bearing mice (Figure 4). These findings also indicate that LDX-mediated alteration of the melanoma-supporting microenvironment additionally attenuates tumor progression and may further contribute to the induction of apoptosis of the malignant cells (eg. C8161).

Presented here studies also support the notion that LDX treatment prevents “rejuvenation’ of the tumors by affecting cells with enhanced tumorigenic capabilities, so called melanoma-initiating cells. Several recent studies demonstrated that a distinct population of human melanoma cells with high ALDH activity is responsible for tumorigenesis and tumor self-renewal [26, 27] and that silencing of ALDH by shRNA leads to melanoma cell cycle arrest, apoptosis, decreased cell viability *in vitro* and reduced tumorigenesis *in vivo* [27]. Therefore, a decrease of the ALDH^+^ melanoma cells in non-apoptotic regions of all LDX-treated tumors (Figure 5) could be directly related to the reduced tumor-regenerating capacity and abrogation of melanoma self-renewal mechanism. Although we identified a higher percentage of the ALDH^+^ cells in the apoptotic regions, ALDH expression coincided with the caspase-mediated cleavage and release of the cytokeratin18 fragments (Figure 5), the hallmark of the intermediate stage of apoptosis. Apoptosis is a tightly regulated process that inevitably leads to structural and biochemical changes and eventually to cell death and phagocytosis. Although, ALDH is an enzyme that detoxifies aldehydic products generated by reactive oxygen species and enhances cell survival, it is unlikely that ALDH expression in apoptotic cells may reverse mid-apoptosis. This notion is indirectly supported by the accumulation of the macrophages in apoptotic regions that phagocytose dead melanoma cells.

Collectively, presented here data demonstrate that Ladarixin, a dual CXCR1/2 small molecule inhibitor, has a multifactorial effect on melanomas *in vitro* and *in vivo* independently of the genetic defects underlying tumorigenic phenotype. These effects include inhibition of melanoma cell motility, NF-kB and AKT-mediated pro-survival signaling, increased apoptosis, down-modulation of pro-angiogenic signaling and formation of the intratumoral microvasculature *de novo*, polarization of the intratumoral macrophages to M1 tumoricidal phenotype, and blocking of the ALDH^+^ cell-dependent melanoma self-renewal mechanism. These pre-clinical findings demonstrated therapeutic utility of LDX for the inhibition of various melanomas independently of the tumor-driving mutations.

## MATERIALS AND METHODS

### Cell lines and culture

All WM and C8161 primary melanoma cell lines were kindly provided by Dr. M.Herlin (Wistar Institute, Philadelphia PA). Cell were cultured in MCDB164/L15 media (4/1) supplemented with 2% Fetal Bovine Serum (FBS), Bovine insulin, and calcium chloride. Uveal melanoma lines UM001 and UM004 characterized by the activating mutations in GNAQ^Q209P^ and GNA11^Q209L^ were provided by Dr. T. Sato (Thomas Jefferson University, Philadelphia PA) and cultured in RPMI1640 media supplemented with 10% FBS, β-mercaptoethanol, penicillin and streptomycin. Derivative melanoma cell lines expressing Nano luciferase under the control of the NF-kB response elements were generated by stable transfection of the parental cells with pNL3.2 NF-kB-RE [NlucP NF-kB-RE Hygro] (Promega, Madison, WI). Primary human melanocytes were kindly provided by Dr. Z. Abdel-Malek (University of Cincinnati, Cincinnati, OH) and cultured in Epilife culture media with TPA-free supplements (Thermo/Fisher, Grand Island, NY).

### Reagents

Ladarixin, a dual CXCR1 and CXCR2 inhibitor was provided by Dompé Farmaceutici SpA, *Via* Campo di Pile, L’Aquila, Italy. LDX was prepared fresh daily by dissolving in culture media (*in vitro* experiments) or in PBS (*in vivo* experiments) to desired concentration.

### Analysis of chemokines secretion

Chemokine-targeted antibody-based array (Ray Biotech, Norcross, GA) was used for analysis of melanocytic cell-derived secreted chemokines as devised by the manufacturer. Normalization and quantitation of chemokine secretion was done using array-specific Ray Biotech Analysis Tool.

### FACS

Fluorescently-labeled CXCR1 and CXCR2-specific antibodies for FACS were from BioLegend (San Diego, CA). Cell sorting was done on Guava EasyCyte system and analyzed using InSyte Software (Millipore, Billerica MA).

### *In vitro* migration assay

Cells were plated at sub-confluent density onto 6-well plates and grown to confluence. Then, a 0.5 mm scratch was introduced and cells were allowed to migrate into the area for 24 h in the presence or absence of LDX (1×10^-5^ M). Ten independent images were taken from each cell line/condition and cells migrated into the scratch, were enumerated.

### Melanoma cell adhesion and attachment

Different melanoma cells were plated onto 96 well plates and allow attaching to plastic substrata for 1, 2, 3 and 4 h or kept up to 5 days in the presence or absence of LDX (1×10^-5^ M) in culture media. At indicated time points, cells were fixed in 3% formaldehyde and stained with 0.1% crystal violet. After washes, plates were scanned at 570 nm on Bio Tech FL600 plate reader (Biotech Inc., Richmond, VA). Results were obtained from three independent experiments, three wells for each experiment.

### Assessment of apoptosis

Apoptosis was assessed using *In Situ* Cell Death detection kit (TUNEL assay) (Roche Bioscience, Indianapolis, IN). Quantitation was done on images of 5 random microscopic fields per cell line per treatment.

### Treatment with LDX *in vitro* and *in vivo*

For all *in vitro* experiments, unless otherwise stated, LDX was dissolved in phosphate buffered saline (PBS), filter-sterilized and used at 1×10^-5^ M. For *in vivo* treatment of tumor-bearing animals, LDX was dissolved in PBS, filter-sterilized and used at 15 mg/kg. LDX was administered *via* intraperitoneal (IP) injection once a day, every day, for the duration of the experiment. The dose was abopted from prior experiments on the 1^st^ generation of CXCR1 inhibitor, Reparixin [40]

### Animal treatment

Eight cohorts of NCrNU-M nude spontaneous mutant standard athymic mice (Taconic, Hudson, NY) (*n* = 10 per cohort) were intradermally injected with 1×10^6^ melanoma cells (WM164, C8161, UM001, UM004) into right flanks in 30µl of saline. Lesions were pre-established for 10 days. Then, experimental animal were treated *via* IP injection of 100 l of LDX in saline at 15 mg/kg dose, whereas 3 cohorts of control mice received saline. Dose and rout of administration were adopted from prior *in vivo* studies with first generation CXCR1 inhibitor, Reparixin [40].

### Tumor measurements

Progression of the intradermal lesions was monitored by measuring 3 longest diameters using digital calipers. Tumor volume was calculated by multiplication of width x length x height.

### Indirect immunofluorescent and Western blot analyses

Indirect immunofluorescent detection of antigens was done on paraformaldehyde fixed, Triton X-100 permeabilized 7 µm cryo-sections or on cells plated onto glass chamber slides (Millipore, Billerica, MA) according to standard protocol. After permeabilization, slides were washed in PBS, blocked with 1% BSA and incubated with primary and secondary antibodies for 1 h each following counterstaining of nuclei with DAPI (Sigma, St. Louis, MO). Species-specific AlexaFluor^488^- or AlexaFluor^594^ - labeled secondary antibodies were from Thermo-Fisher (Thermo/Fisher, Grand Island, NY). Western blot analysis was done using cell and tumor lysates in RIPA buffer. All antibodies were used in recommended dilutions (provided in Supplementary Materials and Methods).

### Quantitation of microvessel density (MVD)

Individual sections of LDX-treated and untreated tumor lesions were stained with CD31-specific antibodies (Thermo/Fisher, Grand Island, NY). Microvessel density was determined on ten random hotspots by direct counting of CD31-positive blood vessels as described previously [41]. Percent inhibition Thermo/Fisher, Grand Island, NY between MVD of untreated and treated lesions. MVD was assessed in control and LDX-treated lesions of a compatible size.

### RT-PCR

Total RNA was isolated from cultured melanoma cells using RNeasy total RNA extraction kit (Qiagen, Valencia, CA). First-strand DNA was synthesized using Super Script III reverse transcriptase (Invitrogen, Grand Island, NY). Chemokines and receptors were amplified using the following primers: CXCL1 forward 5’- AGGGAATTCACCCCAAGAAC-3’ and reverse 5’- TGGATTTGTCACTGTTCAGCA -3’; CXCL8 forward 5’-ATGACTTCCAAGCTGGCC-3’ and reverse 5’-CAGACAGAGCTCTCTTCC-3’; CXCR1 forward 5’- AGGGGCCACACCAACCTTCTG -3’ and reverse 5’- AGTGCCTGCCTCAATGTCTCCA 3’; CXCR2 forward 5’- CAGTTACAGCTCTACCCTGCC -3’ and reverse 5’-CCAGGAGCAAGGACAGACCCC- 3’. Amplification of β-Actin was used as a loading control.

### Quantitation of immunostainings

To quantify cells detected in lesions by the indirect immunofluorescence, 5 consecutive 7 µm cryo-sections were taken from individual samples with 100 µm intervals. All generated sections were stained with respective antibodies and images of 3 random microscopic fields were taken. Fluorescent antibody-labeled cells were quantified on images using ImagePro software. Data for each antigen was averaged and standard deviations were determined.

### Statistical analysis

Comparison of two samples (control *vs* treatment) was done using paired 2-tailed *t*-test. In tumor growth experiments, difference between LDX-treated and control tumors was considered statistically significant when significant difference was observed at 3 consecutive time points. *p* < 0.05 was considered statistically significant.

## SUPPLEMENTARY MATERIALS FIGURES AND TABLES



## Abbreviations

LDX: Ladarixin
AKT: Protein kinase B
NF-kB: nuclear factor kappa B
STAT3: Signal transducer and activator of transcription 3
ALDH: Aldehyde dehydrogenases
TAM: Tumor-infiltrating macrophage
iNOS: inducible Nitric oxide synthases
PBS: Phosphate buffered saline
IP: intraperitoneal
MVD: microvessel density
