# Supplementary material for: Ladarixin, a dual CXCR1/2 inhibitor, attenuates experimental melanomas harboring different molecular defects by affecting malignant cells and tumor microenvironment
Source: Oncotarget. 2017 Jan 24;8(9):14428–42. doi: 10.18632/oncotarget.14803 (PMC5362416; doi:10.18632/oncotarget.14803)
Supplement: Supplementary file 1 [file oncotarget-08-14428-s001.pdf]

Ladarixin, a dual CXCR1/2 inhibitor, attenuates experimental melanomas harboring different molecular defects by affecting malignant cells and tumor microenvironment

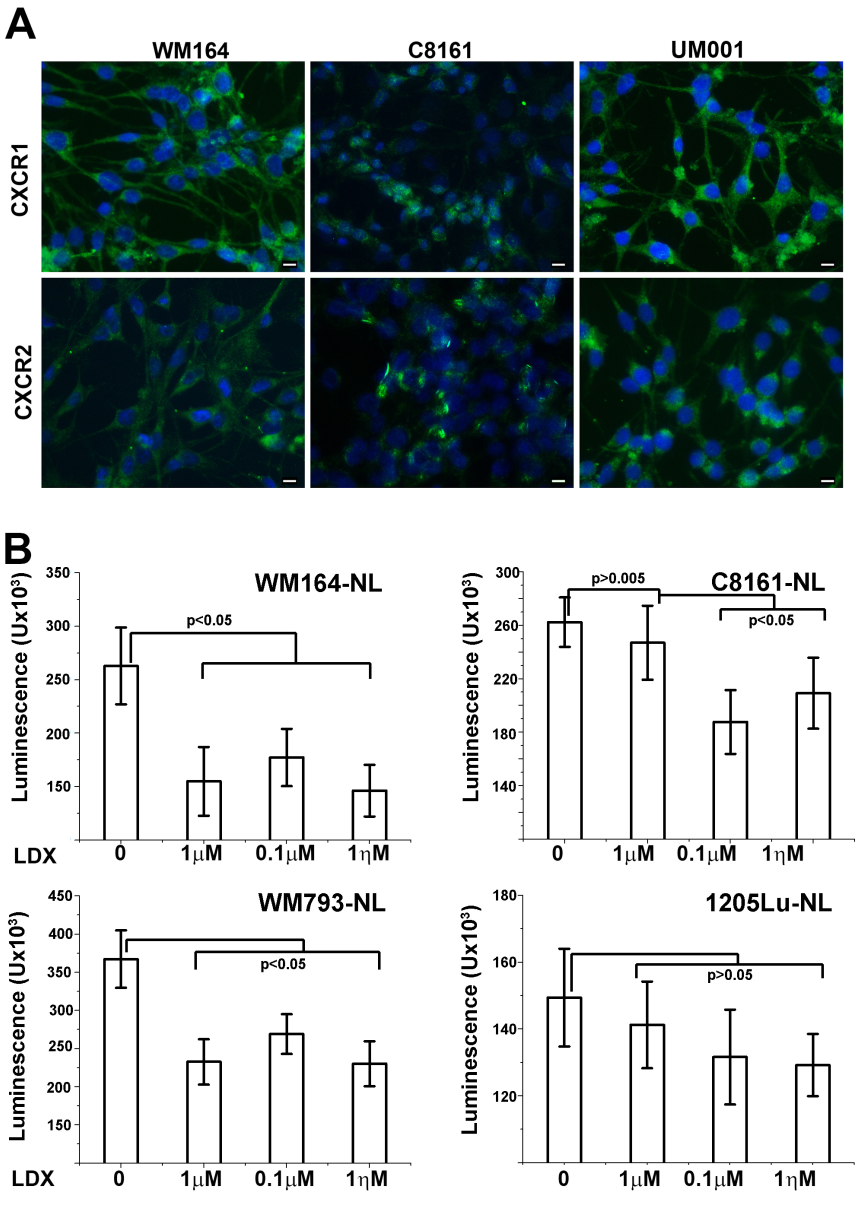

Supplementary Figure S1: Analysis of CXCR1/2 expression and LDX-mediated inhibition of NF-kB activity. **(A) Indirect immunofluorescent detection of CXCR1 and CXCR2 in selected melanoma cell lines (as indicated).** Green – CXCR1/2 receptors, Blue –DAPI nuclear staining. Scale bar – 10μm. Representative cell lines indicated above the panels. Detected receptors are shown to the right of the panels.

(B) Analysis of LDX-mediate inhibition of NF-kB activity in malignant cells expressing NanoLuc under the control of NF-kB response elements. WM164, C8161, WM793 and 1205Lu primary melanoma clines were used to generate derivative NF-kB-NanoLuc lines (NL). NanoLuc activity was measured in 4 independent experiments in triplicates and presented as average units of luminescence ± SD. Statistical significance was assessed by 2-tailed type 2 *t*-test and presented as p-value above the columns. Concentration of LDX is shown below the columns.

Cell lines: Melanoma cells expressing Nano luciferase under the control of the NF-kB response elements were generated by stable nucleofection of cells with pNL3.2 NF-kB-RE [NlucP NF-kB-RE Hygro] (Promega, Madison, WI) plasmid and selection with Hygromycin for 4 weeks. Individual clones were examined for the NF-kB-dependent induction of NanoLuc by exposing cells to TNFα as devised by the manufacturer (Promega, Madison, WI). Individual responding cloneds for each cell type were selected, propagated and used for the studies.

Indirect immunofluorescence: Tissue samples from control and treated lesions were collected, snap-frozen in liquid nitrogen, mounted into the OCT (optimal cutting temperature) compound (T). Then, 7 µm cryo-sections were prepared, fixed with 4 % paraformaldehyde in PBS and blocked with 1% BSA in PBS for 1 h. Incubation in primary and secondary antibodies were done in PBS for 1 h with 4 consecutive washes in PBS between the incubations. After last wash, sections were stained with DAPI (Sigma), washed once in water and mounted with FluoroSafe reagent (EMD Millipore). Species-specific secondary antibodies labeled with AlexaFluor^{488} or AlexaFluor^{594} were from Thermo-Fisher (Life Technologies).

Western Blot : Cells and tissues were lysed in RIPA buffer containing PMSF and Protease/Phosphatase inhibitors cocktail (Thermo Fisher). Lysates were clarified by centrifugation at 10000 x g for 10 min at +4^{0}C. Total protein concentration was measured by colorimetric assay using 660 reagent (Thermo Fisher). Ten micrograms of total protein were loaded per lane and separated on 4-20% SDS-PAGE gels. Proteins were transferred onto PVDF membrane and blocked with 3% BSA in Tris-tween buffered saline (TTBS) following incubation with primary and secondary antibodies (1 h each) with 4 washes in TTBS in between. Detection of immuno-complexes was done using WestFemto chemoluminescent substrate (Thermo Fisher).

Antibodies for indirect immunofluorescent detection and Western Blot analyses:

Antigen	Antibody source	Catalog #	Application	Dilution
CXCR1	GeneTex	GTX100389	WB / IF-Fr	1:2000 / 1:100
CXCR1-PE	BioLegend	32608	FACS	1:50
CXCR2	Bioss Ink	Bs-1629R	WB / IF-Fr	1:2000 / 1:100
CXCR2-AlexaFluor^{488}	BioLegend	320712	FACS	1:50
CD11b	BD Parmingen	557394	IF-Fr	1:200
Cleaved PARP	Cell Signaling	9541	IF-Fr	1:100
Ki-67	Abcam	ab15580	IF-Fr	1:100

iNOS	BD Transduction Labs	610329	IF-Fr	1:100
CD31	ThermoFisher	RB-10333-P0	IF-Fr	1:200
M30 CytoDEATH	Roche Bioscience	12140322001	IF-Fr	1:200
ALDH			IF-Fr	1:100
NF-kB	Abcam	ab7970	WB / IF-Fr	1:2000 / 1:100
P^{Ser536}-NF-kB	Cell Signaling	3033	WB	1:2000
AKT	Cell Signaling	9272	WB	1:2000
P^{Thr308}-AKT	Cell Signaling	9275	WB	1:2000
STAT3	Cell Signaling	9139	WB	1:2000
P^{Tyr705}-STAT3	Cell Signaling	9145	WB / IF-Fr	1:2000 / 1:100
E-Cadherin	Affimetrix/eBioscience	14-3259	WB	1:5000
N-Cadherin	Affimetrix/eBioscience	14-3249	WB	1:5000
P-Cadherin	Affimetrix/eBioscience	14-9873	WB	1:5000
ALDH	Abcam	Ab52492	IF-Fr	1:100

Assessment of NF-kB-dependent NanoLuc activity: All generated melanoma lines expressing NanoLuc under the control of the NF-kB response element were tested for the NF-kB-dependent activation of the luciferase by standard TNFα induction assay as devised by the manufacturer of the pNL3.2 NF-kB-RE plasmid (Promega, Madison, WI). To assess LDX-dependent expression of the NanoLuc, cells were treated with different concentration of LDX for 24 h. Then, luciferase activity was measure using the Nano-Glo® Luciferase Assay System as devised by the manufacturer (Promega, Madison, WI).
